# Empirical mode decomposition based long short-term memory neural network forecasting model for the short-term metro passenger flow

**DOI:** 10.1371/journal.pone.0222365

**Published:** 2019-09-11

**Authors:** Quanchao Chen, Di Wen, Xuqiang Li, Dingjun Chen, Hongxia Lv, Jie Zhang, Peng Gao

**Affiliations:** 1 School of Transportation and Logistics, Southwest Jiaotong University, Chengdu, China; 2 National Railway Train Diagram Research and Training Center, Southwest Jiaotong University, Chengdu, China; 3 National and Local Joint Engineering Laboratory of Comprehensive Intelligent Transportation, Southwest Jiaotong University, Chengdu, China; Tongii University, CHINA

## Abstract

Short-term metro passenger flow forecasting is an essential component of intelligent transportation systems (ITS) and can be applied to optimize the passenger flow organization of a station and offer data support for metro passenger flow early warning and system management. LSTM neural networks have recently achieved remarkable recent in the field of natural language processing (NLP) because they are well suited for learning from experience to predict time series. For this purpose, we propose an empirical mode decomposition (EMD)-based long short-term memory (LSTM) neural network model for predicting short-term metro inbound passenger flow. The EMD algorithm decomposes the original sequential passenger flow into several intrinsic mode functions (IMFs) and a residual. Selected IMFs that are strongly correlated with the original data can be obtained via feature selection. The selected IMFs and the original data are integrated into inputs for LSTM neural networks, and a single LSTM prediction model and an EMD-LSTM hybrid forecasting model are developed. Finally, historical real automatic fare collection (AFC) data from metro passengers are collected from Chengdu Metro to verify the validity of the proposed EMD-LSTM prediction model. The results indicate that the proposed EMD-LSTM hybrid forecasting model outperforms the LSTM, ARIMA and BPN models.

## Introduction

With the development of cities and aggregation of population, the problem of public transport travel is becoming increasingly important. Urban rail transit has become the first choice for mass transit travel because of its large capacity, high speed and safety. In recent years, the urban metro has developed rapidly but in the pursuit of “quality” metro travel by the public, such issues as congestion, transfer inconvenience and other issues must be urgently solved.

Based on the time horizon of prediction, metro passenger flow prediction can be broadly classified into three different classes: long-term forecasting, medium-term forecasting and short-term forecasting. Medium-term and long-term passenger flow forecasting usually have a forecasting range of the next 3 to 15 years, and short-term passenger flow forecasting generally has a forecasting range of the next week or month.

The study of urban metro passenger flow is a key aspect of metro planning, operation and safety protection application. Short-term metro passenger flow prediction can be applied to optimize the lines and passenger flow organization of the stations, supply early warnings for the operators and multi-party decision-making for public travel, and further improve the scientific management level of the urban metro, which is of practical importance to safety and operation. The prediction can offer evidence for an advanced approach to passenger flow control and induction, which can prevent congestion, trampling and other safety accidents. Therefore, accurate prediction of the short-term metro passenger flow is of great significance.

Short-term metro passenger flow consists of non-linear and non-stationary time series data, and because many factors influence passenger flow, it is difficult to extract and quantify them, which is the most challenging issue. As a result, the research on short-term passenger flow prediction has become an active study topic.

Currently, theoretical research on passenger flow forecasting models can be roughly divided into four categories:

1. Non-linear models, including non-parametric regression [1], chaos theory [2,3], grey theory [4–6], neural network [7–9], wavelet network [10], and support vector machine (SVM) [11,12];
2. Linear models, including time series models [13,14] and state space models [15];
3. Combined models in which the above models are combined, including algorithm optimization [16,17], filtering [18,19], model fusion [20–23], etc.
4. Other models, such as simulation models [24].

In other forecasting areas, the researchers in [25] developed an unbalanced panel data mixed logit model to predict the crash likelihood, and the results showed its strong versatility in handling a large amount of data, which can be leveraged in future big data applications in transportation. A multivariate space-time modeling framework was proposed within a full Bayesian paradigm to analyze crash frequency [26], and the mixed logit model was adopted to analyze the injury severity of truck drivers on rural highways [27]. In [28], researchers proposed a square support vector machine with hybrid optimization algorithm for short-term traffic flow prediction.

However, traditional time series prediction models such as the ARIMA model encounter difficulty in accurate calibration of parameters, and they must be re-fitted when new passenger flow data are obtained. Additionally, the generalization ability of the models is weak. Grey theory prediction models are designed to accumulate production time series of historical data, which eliminates the fluctuation and randomness of the short-term passenger flow to a certain extent. It is difficult for the support vector machine (SVM) to train a large number of passenger flow data.

With the development of deep learning, artificial neural networks have attracted much attention. In the book *Neural Networks and Deep Learning*, Michael Nielsen proved that neural networks can approximate any function. Neural networks have good self-adaptability, self-organization and strong learning ability, and they are more suitable for passenger flow prediction with non-linear and more complex data. However, for traditional neural networks such as back-propagation neural networks (BPN), which are a local search optimization method, it is easy for the algorithm to fall into local extrema, and the slow convergence speed of the algorithm makes BPN insufficiently accurate for short-term metro passenger flow prediction. Researchers have proposed various new deep neural network models in the last several years. Deep belief neural networks (DBNN) [29], the sparse autoencoder (SAE) [30], convolution neural networks (CNN) [31], and graph convolution networks (GCN) [32] are also used in short-term passenger flow prediction and have achieved good performance.

Recurrent neural networks (RNN) introduce the concept of sequence, which makes them better able to process time series data. RNNs have achieved remarkable results in the field of natural language processing (NLP), such as machine translation and speech recognition.

However, due to the “gradient vanishing” problem in RNN, Hochreiter & Schmidhuber proposed an improved RNN network in 1997 [33], namely, the long short-term memory (LSTM) neural network, which adds a "gate" structure to the RNN network structure and mitigates the vanishing gradient problem using a “forget gate” and “output gate” that can allow the information to pass selectively. Unlike traditional RNNs, the LSTM neural networks are well suited for learning from experience to predict time series when long time lags occur in large-scale historical data. The work in [34,35] indicates that LSTM neural networks are suitable for short-time passenger flow prediction.

Empirical mode decomposition (EMD), as the first portion of the Hilbert-Huang transform (HHT) introduced by Huang et al. in 1998, is used in analyzing non-linear and non-stationary time series data [36,37]. The EMD method is able to decompose the original data into several intrinsic mode functions (IMFs) via a sifting process, which does not need to preset any basis functions. Because it can address non-linear and non-stationary data, certain researchers developed hybrid short-term passenger flow prediction models based on EMD. For example, in [38], a hybrid EMD-BPN forecasting approach was developed to predict the short-term metro passenger flow and suggested that the proposed hybrid EMD-BPN approach performed well and stably in forecasting the short-term metro passenger flow. In [39], an empirical mode decomposition (EMD)-based recurrent Hermite neural network prediction model was proposed for highly non-linear and non-stationary short-term traffic flow, and it achieved superior performances compared with the pure recurrent neural network and RHNN models.

Considering that the short-term metro inbound passenger flow consists of non-linear and non-stationary time series data, the EMD method can be applied to analyze the non-linear and non-stationary data, and the LSTM neural networks are well suited for prediction of time series by learning from historical data. An EMD-LSTM hybrid prediction model could offer a new approach to accurate prediction of the short-term metro passenger flow.

In this paper, we attempt to extract the data characteristics of the influencing factors for passenger flow fluctuation by applying the EMD method to decompose the historical data. An empirical mode decomposition (EMD)-based long short-term memory (LSTM) neural network model is constructed to predict the short-term metro inbound passenger flow. The main contribution of this paper is that the feasibility and validity of the EMD-LSTM hybrid forecasting model is verified. Moreover, this approach can effectively improve the accuracy of the short-term metro inbound passenger flow prediction.

The remainder of our paper is structured as follows. First, the EMD method and the LSTM neural network theories are briefly described. Second, the framework of a single LSTM model and the EMD-LSTM hybrid model is shown. Third, a case study is proposed, and the results are analyzed. Finally, the limitations of this paper are discussed, and selected conclusions are drawn.

## Methodology

### Empirical mode decomposition

Empirical mode decomposition (EMD) is an adaptive signal analysis method proposed by Huang et al. in 1998 to address non-linear and non-stationary signals. EMD can decompose the non-linear and non-stationary data into a finite and small number of different intrinsic model functions (IMFs), which are based on the local characteristic time scale of the data. Each IMF is a signal sequence with a different oscillation mode and can reflect certain physical meanings of the original signal, but it must meet the following two conditions:

1. The number of extrema and the number of zero crossings must be equal or differ at most by one in the whole data set;
2. The mean value of the envelope defined by the local maxima and the envelope defined by the local minima is equal to zero at any point.

The process of EMD is indeed a sifting process. The algorithm of EMD is described as follows:

Step 1. Identify all local extrema in the original data x(t), including the minimum value min(t) and maximum value max(t).
Step 2: Generate the upper envelope of all local maxima e_max_(t) and the lower envelope of all local minima e_min_(t) using a cubic spline line.
Step 3: Calculate the mean m_1_(t) of e_max_(t) and e_min_(t):
  $${\text{m}}_{1}(\text{t})=\frac{\left({\text{e}}_{\text{max}}(\text{t})+{\text{e}}_{\text{min}}(\text{t})\right)}{2}$$ (1)
Step 4: The first intrinsic mode function h_1_(t) is defined as
  $${\text{h}}_{1}(\text{t})=\text{x}(\text{t})-{\text{m}}_{1}(\text{t})$$ (2)
Step 5: Check whether h_1_(t) meets the above two conditions. If it does, then h_1_(t) is denoted as the first IMF of x(t), and residue r_1_(t) is substituted for the original data x(t),
  $${\text{r}}_{1}(\text{t})=\text{x}(\text{t})-{\text{h}}_{1}(\text{t})$$ (3)
  Otherwise, replace the original data x(t) with h_1_(t).
Step 6: Repeat the above steps. The sifting process stops when the residue becomes a monotonic function, a constant value, or a function with only one extremum such that no more IMF can be extracted.

As a result, the original data x(t) can be expressed as:

$$\text{x}(\text{t})=\sum_{\text{i}=1}^{\text{n}}{\text{c}}_{\text{i}}(\text{t})\text{+r}(\text{t})$$ (4)

where c_1_(t), c_2_(t), …, c_n_(t) are the *1*th, *2*th, …, *n*th IMF, respectively, of the original data x(t), and the final residue r(t) represents a trend of the original data.

### Long short-term memory neural networks

Long short-term memory (LSTM) neural networks are a special type of the recurrent neural networks (RNN) [40]. The calculation results of each hidden layer in the RNN are related to the result of the current input and the last hidden layer, which means that the output of the previous moment has a direct impact on the input of the current moment. Compared with other neural networks, RNN could be more suitable to addressing time series data due to its ability to use contextual information when mapping between input and output sequences. However, it is not proper for RNN to address the long-term dependencies of data because of the problem of vanishing gradients in RNN [41].

**Fig 1:**
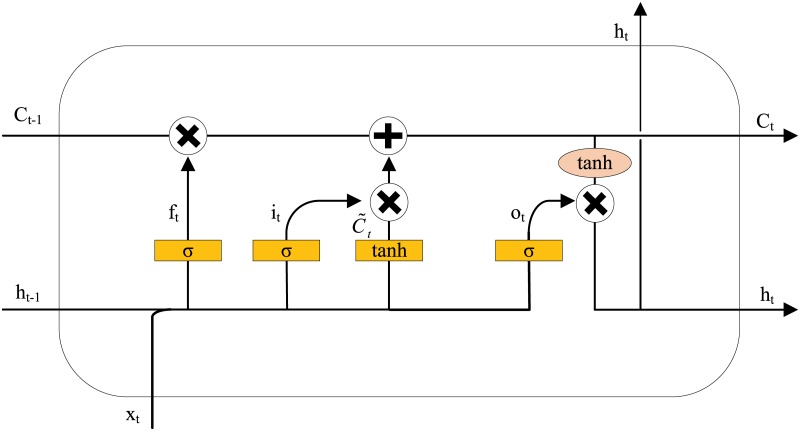
LSTM memory block.

The LSTM network was proposed by Hochreiter & Schmidhuber to overcome the problem of vanishing gradients with the use of gates (forget gate, input gate and output gate) that can retain information selectively. Different from a standard RNN, the LSTM model introduces a memory block, which is shown in Fig 1 below. The mechanism of the gates is summarized briefly as follows.

The gate activation function σ is usually the logistic sigmoid such that the gate activations are between 0 (gate closed) and 1 (gate open). There are three inputs to the LSTM neural network: x_t_ is the input at time t, h_t-1_ is the output of the memory block at time t-1, and C_t-1_ is the state of memory cell at time t-1. There are two outputs: h_t_ is the output of the memory block at time t, and C_t_ is the state of memory cell at time t. Additionally, W_f_, W_i_, W_o_, W_c_ are weight matrices, and b_f_, b_i_, b_o_, b_c_ are bias vectors.

1. Forget gate
  The forget gate reads the current input *x*_t_ and the previous output of memory block h_t-1_. How much information to retain from the upper cell is decided by the activation function σ of the forget gate.
  $${\text{f}}_{\text{t}}=\sigma ({\text{W}}_{\text{f}}∙[{\text{h}}_{\text{t}-1},{\text{x}}_{\text{t}}]+{\text{b}}_{\text{f}})$$ (5)
2. Input gate
  The input gate controls how much information can flow into the cell and is obtained by the activation function σ, expressed as:
  $${\text{i}}_{\text{t}}=\sigma ({\text{W}}_{\text{i}}∙[{\text{h}}_{\text{t}-1},{\text{x}}_{\text{t}}]+{\text{b}}_{\text{i}})$$ (6)
3. Output gateThe output gate can regulate the output of the memory cell.
  $${\text{o}}_{\text{t}}=\sigma ({\text{W}}_{\text{o}}∙[{\text{h}}_{\text{t}-1},{\text{x}}_{\text{t}}]+{\text{b}}_{\text{o}})$$ (7)

The state of the new cell and old cell is updated at time t from C_t-1_ to C_t_,

$${\tilde{\text{C}}}_{\text{t}}=\text{tanh}\left({\text{W}}_{\text{c}}∙[{\text{h}}_{\text{t}-1},{\text{x}}_{\text{t}}]+{\text{b}}_{\text{c}}\right)$$ (8)

$${\text{C}}_{\text{t}}={\text{f}}_{\text{t}}*{\text{C}}_{\text{t}-1}+{\text{i}}_{\text{t}}*{\tilde{\text{C}}}_{\text{t}}$$ (9)

$${\text{h}}_{\text{t}}={\text{o}}_{\text{t}}*\text{tanh}\left({\text{C}}_{\text{t}}\right)$$ (10)

## Procedural framework

### LSTM prediction model

First, data preprocessing of the original time series metro passenger flow is conducted. Second, data sets suitable for the inputs of the LSTM neural network are constructed via exploratory data analysis (EDA). The LSTM prediction model can be obtained by optimizing the hyperparameters of the LSTM neural network. Finally, the short-term metro passenger flow prediction is made using the LSTM neural network.

### EMD-LSTM prediction model

This paper decomposes the preprocessed data via the EMD method, and as such, several IMF components and Res can be obtained. We integrate the selected IMF components with the preprocessed data to construct the data sets. Hence, the EMD-LSTM hybrid prediction model is developed, and the results of the EMD-LSTM hybrid prediction model can be obtained.

**Fig 2:**
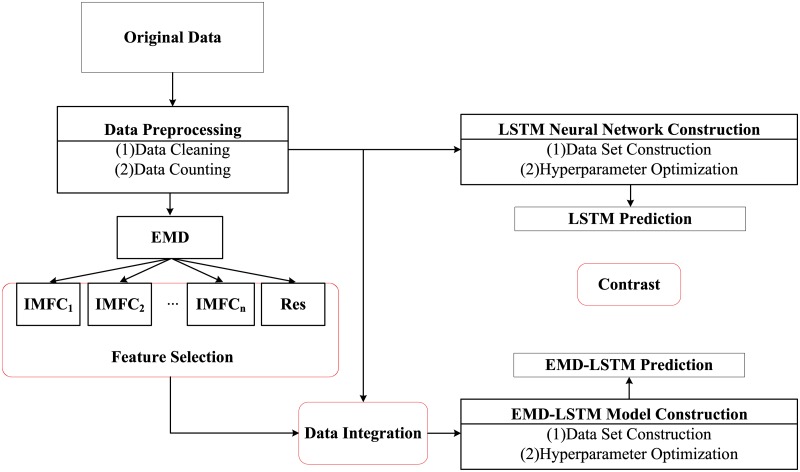
Schematic diagram.

The schematic diagram of this paper is illustrated in Fig 2.

## Case study

### Data preprocessing

The original data are collected from the automated fare collection (AFC) system in Chengdu Metro. The inbound passenger flow data of Xipu station from 06:00 to 22:00 are selected for three consecutive weeks, from April 9 to April 29, 2018. First, we preprocess the raw data, including data cleaning and data counting. The rules for data cleaning are given as follows:

1. Delete the completely duplicated records;
2. Delete the records with missing values;
3. Delete the records with the same inbound and outbound station;
4. Delete the records with inbound time later than outbound time;
5. Delete the records with travel times that are too long or too short, where the travel time is equal to the subtraction of the inbound time from the outbound time.

Based on the preprocessed data, the number of inbound passengers of XiPu station is counted with a frequency of 15 min. As a result, 64 observations are obtained within a day, and the preprocessed data contain 1344 observations.

### Problem statement

We attempt to predict the number of inbound passengers of XiPu station on April 27 based on historical data, namely, to predict the 64 observations on April 27 (Friday).

In the following section, first, we construct the data sets via EDA. Second, a single LSTM prediction model and an EMD-LSTM hybrid prediction model are proposed to predict the 64 observations. The two baseline models (ARIMA prediction model and BPN prediction model) are used to compare the above two models. Finally, the EMD-LSTM hybrid prediction model with the best performance can be verified.

### Exploratory data analysis

**Fig 3:**
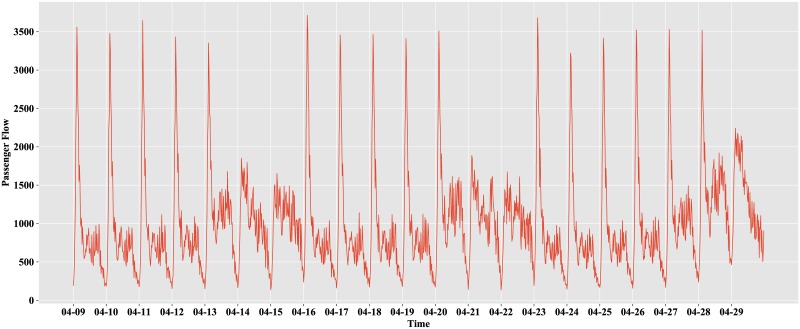
Inbound passenger flow.

The preprocessed data range from April 9 to April 29, i.e., three consecutive weeks from Monday to Sunday. A visualization of the preprocessed data is shown in Fig 3.

Based on the above figure, we note that the inbound passenger flow has a certain periodicity. The distributions of the inbound passenger flow on weekdays (Monday to Friday) and weekends (Saturday and Sunday) are inconsistent. The distribution of inbound passenger flow on weekdays is highly similar for the three consecutive weeks. The distribution of inbound passenger flow on weekends is highly similar for the three consecutive weeks.

**Fig 4:**
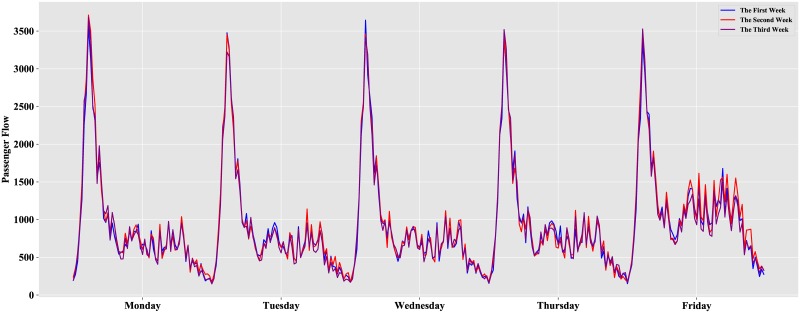
Inbound passenger flow on weekdays.

Because we attempt to predict the 64 observations on April 27, namely, the Friday of the third week, this paper focuses only on the inbound passenger flow on weekdays. We eliminate the passenger flow on weekends and observe the remaining data, as shown in Fig 4.

**Fig 5:**
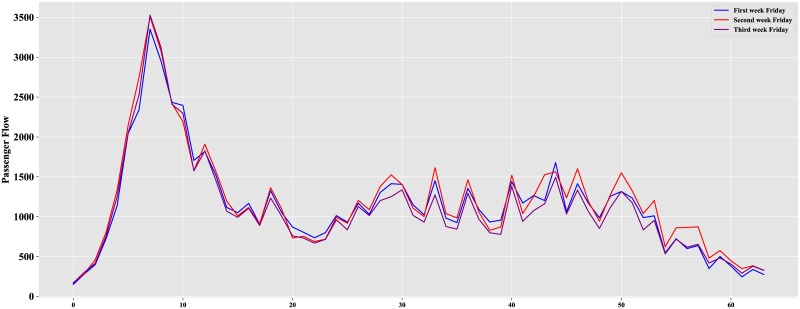
Inbound passenger flow on Friday.

As described in Fig 4, a peak of passenger flow occurs on weekdays. The distribution from Monday to Friday is strongly correlated for three consecutive weeks. The fluctuations of passenger flow from Monday to Thursday are highly similar, but they are different from those of Friday. The inbound passenger flow on Friday is illustrated in Fig 5.

As shown, the Friday of the third week and the Fridays of first two weeks have a strong correlation, which can be used to construct data sets. Usually, the inbound passenger information of the previous day can also make a difference to the next day. Based on this observation, this paper constructs data sets by integrating the data of the same day two weeks ago, the same day of last week, and the previous day.

### Feature selection

**Fig 6:**
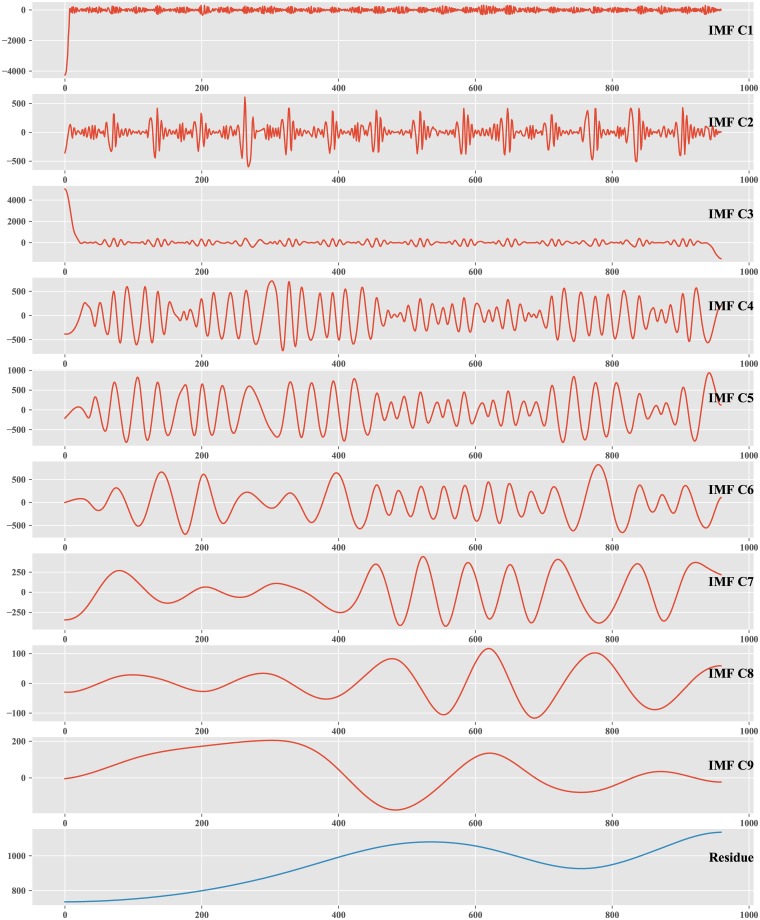
IMFs and Res.

Nine IMF components (IMF C1, IMF C2, …, IMF C9) and one residue are obtained by decomposing the passenger flow data of the first two weekdays by EMD, as shown in Fig 6.

All of the extracted IMF components are illustrated in the order shown in Fig 6. The order of IMFs can reflect the frequency from the highest to the lowest. IMF C1, with the highest frequency, represents the high time variant or noise of the original data, and the residue with the lowest frequency represents the long period component, which is the trend of the original data.

The ten components might be strongly or weakly correlated with the original data. In the feature selection stage, the Spearman correlation coefficient and Kendall correlation coefficient, which are suitable for data that are not normally distributed, are introduced to measure the correlation between each IMF component and the original data and to filter the components that are strongly related to the original data.

In this paper, the Spearman correlation coefficient and Kendall correlation coefficient are comprehensively considered. The Spearman correlation coefficient and Kendall correlation coefficient of each IMF component and the original data are calculated and are summarized in Table 1.

**Table 1:** Correlation coefficients.

IMF	Spearman Correlation Coefficient	Kendall Correlation Coefficient
IMF C1	0.206597	0.147281
IMF C2	0.079371	0.064673
IMF C3	0.278429	0.193597
IMF C4	0.286980	0.199021
IMF C5	0.459662	0.316910
IMF C6	0.352146	0.231368
IMF C7	0.166216	0.107055
IMF C8	0.092003	0.059661
IMF C9	0.109719	0.072157
Res	0.058627	0.036630

From Table 1, the Spearman correlation coefficients of IMF C1 and IMF C3-C7 are, respectively, 0.21, 0.28, 0.29, 0.46, 0.35, and 0.17, indicating a stronger correlation with the original than other IMFs. Observing the Kendall correlation coefficients, those of IMF C1 and IMF C3-C7 are 0.15, 0.19, 0.20, 0.32, 0.23, and 0.11, respectively, which have higher ranks. Therefore, the Spearman correlation coefficients and Kendall correlation coefficients of IMF C1 and IMF C3-C7 are consistent. For this reason, IMF C1 and IMF C3-C7 are selected to construct the data sets.

### Model design

This paper does not consider external factors (such as weather, passenger flow at adjacent stations, etc.), and establishes a deep learning passenger flow prediction model based on pure historical data. The data set of the model is constructed as follows.

We denote x_t,it,i_ as the passenger flow of the *i*th time period on the *t*th day (the inbound passenger flow is counted with a frequency of 15 min, and 64 observations are obtained within a day), x_t-1,i_ is the passenger flow of the *i*th time period on the *t-1*th day, x_t-7,i_ is the passenger flow at the same time period in the previous week, and x_t-14,i_ is the passenger flow at the same time period two weeks ago. In addition, six time series data with the same length as the original data can be obtained by exporting the selected IMFs.

**Fig 7:**
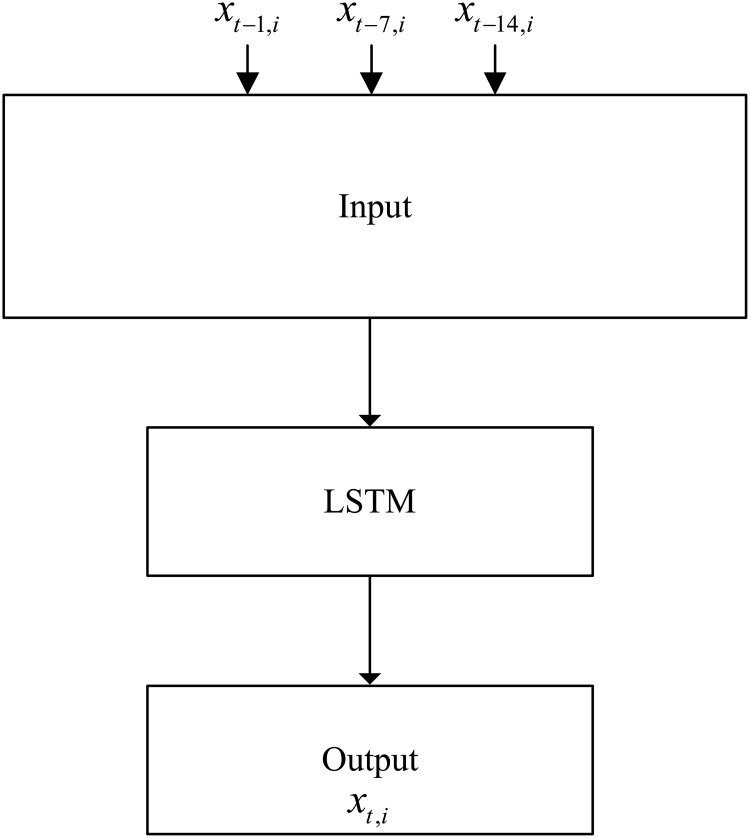
LSTM.

**Fig 8:**
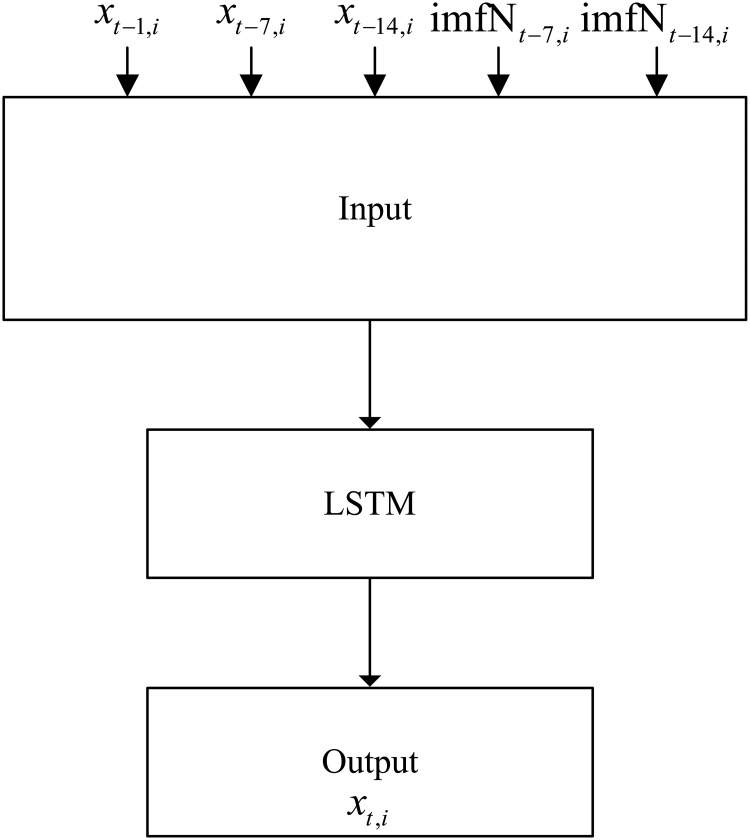
EMD-LSTM.

We define imfN_t-14,i_ (N = 1,3,4,5,6,7) as the value of the *N*th IMF at the same time period two weeks ago, and imfN_t-7,i_ as the value of the *N*th IMF at the same time period in the previous week. The data from the first two weeks are used as the input of the model, and the data from the third week are used as the output of the model to construct a deep neural network model with supervised learning. The data sets construction for a single LSTM prediction model and an EMD-LSTM hybrid prediction model are designed as depicted in Figs 7 and 8.

There are 3 features for the single LSTM model and 15 features for the EMD-LSTM model.

## Results

The data on the three consecutive weeks (no weekends included) are divided into a training data set and test data set. The training set contains the inbound passenger flow data for 14 days, which is fed into the LSTM neural network to optimize hyperparameters of the model. The test set is the last day (Friday), which is applied to validate the effectiveness of forecasting models. Considering the large gap between the peak passenger flow and small passenger flow, we use MinMaxScaler of the Sklearn Library in Python to scale the passenger flow data in the range (0, 1) for both the training set and test set. The predicted values are re-scaled to compare with the real values during validation.

**Fig 9:**
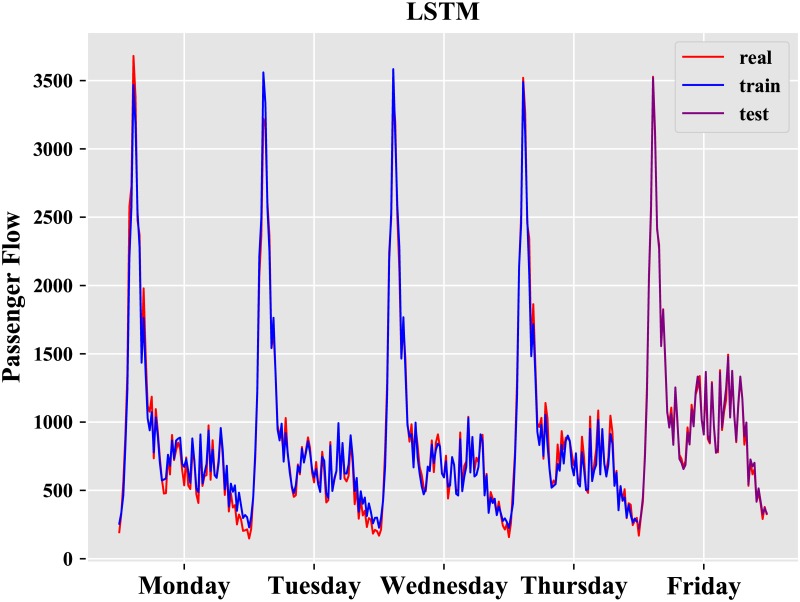
Result of LSTM.

**Fig 10:**
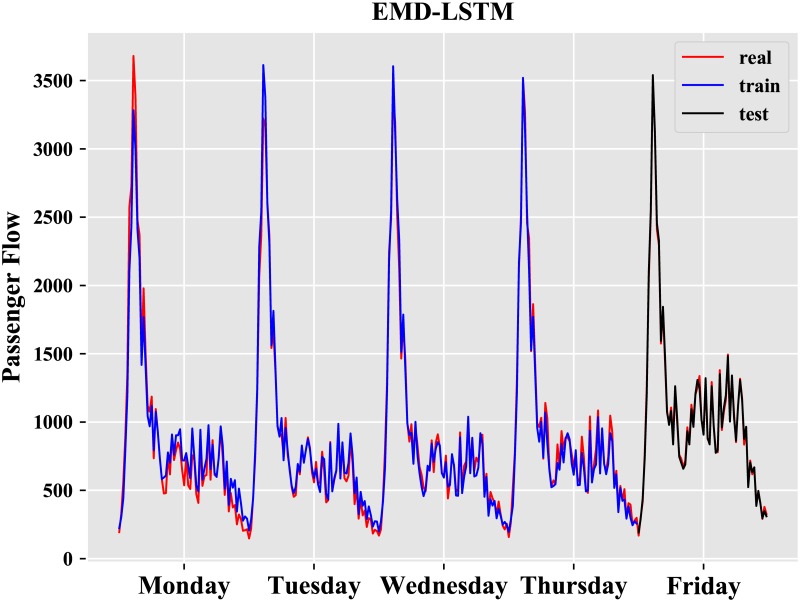
Result of EMD-LSTM.

The entire experiment is completed in the Python Keras Library. The optimizer of the LSTM models is Adam. The performance metrics applied in the study are root mean square error (RMSE) and mean absolute error (MAE).

$$\text{RMSE}=\sqrt{\frac{1}{\text{N}}\sum_{\text{i}=1}^{\text{N}}{\left({\text{y}}_{\text{i}}-{\hat{\text{y}}}_{\text{i}}\right)}^{2}}$$ (11)

$$\text{MAE}=\frac{1}{\text{N}}\sum_{\text{i}=1}^{\text{N}}\left|{\text{y}}_{\text{i}}-{\hat{\text{y}}}_{\text{i}}\right|$$ (12)

where y_i_ is the real value, and ${\hat{\text{y}}}_{\text{i}}$ is the predicted value. The results of the best model obtained after repeated experiments are described in Figs 9 and 10.

In addition, we build two other baseline models, ARIMA and back-propagation neural network (BPN), for comparison with the single LSTM model and the EMD-LSTM hybrid model. The parameters of ARIMA (p, d, q) are optimized through a grid search, and the result of the best BPN model is obtained as shown in Table 2. The p, d, and q parameters in this paper are 2, 1, and 7, respectively. The RMSE and MAE in the training set and test set are summarized in Table 2.

**Table 2:** Comparison of 4 models.

Model	trainRMSE	trainMAE	testRMSE	testMAE
ARIMA (2,1,7)	58.246	45.648	50.659	43.531
BPN	45.963	39.291	41.278	35.242
LSTM	39.209	32.934	36.246	28.164
EMD+LSTM	33.353	26.149	30.466	24.193

**Fig 11:**
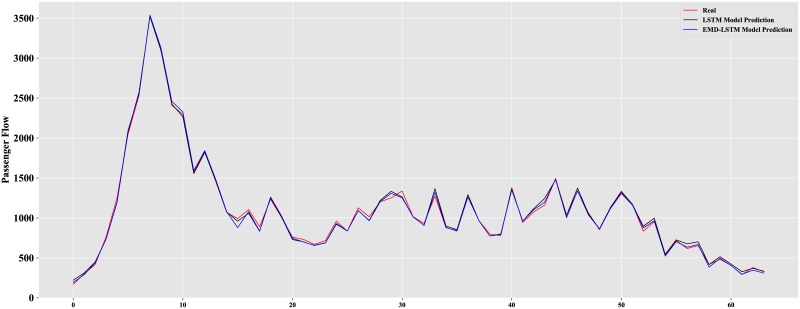
Comparison of LSTM and EMD-LSTM model.

From Table 2, it is obvious that the EMD-LSTM model has the best performance whether on the training set or the test set, and the performance of LSTM model is better than that of ARIMA and BPN. We further analyze the differences between the EMD model and EMD-LSTM model. The results of the LSTM prediction model and the EMD-LSTM prediction model on Friday are described in Fig 11.

There are 64 observations on Friday from 6:00 to 22:00 with an interval of 15 min. From Table 2, we observe that the LSTM neural network is well suited for forecasting the short-term metro passenger flow and is better than the BPN model and ARIMA model.

From Fig 11, the prediction curve of the EMD-LSTM model fits better at many points and is closer to the real value than the LSTM model. The MAE in the test set of the EMD-LSTM model is lower than the MAE of the LSTM model, 24.193 and 28.164, respectively. Obviously, the proposed EMD-LSTM hybrid prediction model achieves the best performance for short-term metro passenger flow prediction.

## Discussion

The theoretical feasibility of the EMD-LSTM hybrid prediction model lies in the following two points. First, the short-term metro inbound passenger flow, which is non-linear and non-stationary, can be decomposed into several IMF components by the EMD method. Thus, IMFs strongly correlated with the original data by feature selection as new features can be added to the inputs of the LSTM model. Second, the LSTM neural network is well suited to prediction of time series data and has achieved remarkable results in the field of natural language processing (NLP). In theory, an EMD-LSTM hybrid forecasting model can be better than a single LSTM forecasting model for short-term metro passenger flow prediction. Finally, the results of our research verify the feasibility and validity of the EMD-LSTM hybrid forecasting model, which is similar to those reported earlier [30,38].

The limitations of this paper are clear. First, there are insufficient data to verify the validity of the model, leading to a slightly larger value of MAE. Another limitation is the characteristic of Xipu metro station. Xipu metro station is the starting station of Chengdu Metro Line 2, and it is a transfer station between the suburban railway and urban rail transit, which share the same platform, a rare occurrence in China. This station is a distribution center with large passenger flow, and the short-term passenger flow is prone to large fluctuations. Therefore, it is difficult to predict the short-term passenger flow accurately.

Second, although a finite set of IMFs can be generated by EMD, these IMFs lack the theoretical fundamentals for explaining the physical meanings. Generally, it is necessary to interpret the meanings of the extracted IMFs with domain knowledge. Third, this paper aims to establish a purely data-driven deep learning passenger flow prediction model, and external factors (weather and holidays, etc.) and spatial characteristics (adjacent stations, etc.) are not considered [42]. Therefore, the above points can be used as directions for future research.

## Conclusion

An EMD-LSTM hybrid model for predicting short-term metro inbound passenger flow is developed in this paper. The results of this paper verify the feasibility and validity of the EMD-LSTM hybrid forecasting model. The major contributions of this paper are as follows:

1. This paper demonstrates that the LSTM neural network is well suited for forecasting short-term metro passenger flow;
2. The EMD-LSTM hybrid prediction model performs better than the single LSTM prediction model in regard short-term metro passenger flow prediction and is the best model in our experiments.

In general, the EMD-LSTM model can effectively improve the accuracy of short-term metro passenger flow prediction.
