# Correction: Empirical mode decomposition based long short-term memory neural network forecasting model for the short-term metro passenger flow

**DOI:** 10.1371/journal.pone.0231199

**Published:** 2020-03-26

**Authors:** Quanchao Chen, Di Wen, Xuqiang Li, Dingjun Chen, Hongxia Lv, Jie Zhang, Peng Gao

## Notice of Republication

This article was republished on March 5, 2020, to remove a Supporting Information file that was incorrectly included in the originally published article. The article’s Data Availability statement has also been updated to reflect this change. Please download this article again to view the correct version.
